# Understanding the P×S Aspect of Within-Person Variation: A Variance Partitioning Approach

**DOI:** 10.3389/fpsyg.2015.02004

**Published:** 2016-01-26

**Authors:** Brian Lakey

**Affiliations:** Psychology, Grand Valley State University, AllendaleMI, USA

**Keywords:** Person × Situation, P×S, SRM, RRT, G theory, within-person variation

## Abstract

This article reviews a variance partitioning approach to within-person variation based on Generalizability Theory and the Social Relations Model. The approach conceptualizes an important part of within-person variation as Person × Situation (P×S) interactions: differences among persons in their profiles of responses across the same situations. The approach provided the first quantitative method for capturing within-person variation and demonstrated very large P×S effects for a wide range of constructs. These include anxiety, five-factor personality traits, perceived social support, leadership, and task performance. Although P×S effects are commonly very large, conceptual, and analytic obstacles have thwarted consistent progress. For example, how does one develop a psychological, versus purely statistical, understanding of P×S effects? How does one forecast future behavior when the criterion is a P×S effect? How can understanding P×S effects contribute to psychological theory? This review describes potential solutions to these and other problems developed in the course of conducting research on the P×S aspect of social support. Additional problems that need resolution are identified.

This article describes a variance partitioning approach to within-person variation based on Generalizabilty (G) Theory (Cronbach et al., 1972) and the Social Relations Model (SRM; Kenny et al., 2006; Kenny, unpublished computer program). G Theory and the SRM are closely related and can be treated as variations of the same approach for the purposes of this article. The approach defines within-person variation as differences among persons in their profiles of reactions to the same situations, beyond (1) the person’s trait-like tendency to respond in the same way on average, to all situations, and (2) the situation’s tendency to evoke the same response, on average, across people. The approach has revealed very large P×S effects for a wide range of constructs, including anxiety (Endler and Hunt, 1966, 1969), five-factor traits (Van Heck et al., 1994; Hendriks, 1996), leadership (Livi et al., 2008; Kenny and Livi, 2009), social support (Lakey and Orehek, 2011) and task performance (Woods et al., in press).

Yet, the approach has not reached its full potential because of conceptual and analytic challenges, as investigators seem to have trouble moving beyond estimating the strength of P×S effects. One commonly sees a few studies showing strong P×S effects and no further progress. This stunted progress leaves many important questions unposed and unanswered. For example, what is the psychological meaning of P×S effects and how is this different from the effects of personality traits and situations? How does one conduct research to reveal this psychological meaning? Can P×S effects forecast important outcomes (e.g., leadership or job performance)? What research designs are appropriate for such forecasting? How can understanding P×S effects inform psychological theory? This article describes proposed solutions to many of these questions by drawing from recent P×S research on social support and identifies additional problems to be solved. This article will focus on conceptual issues rather than on statistical procedures. There are many excellent sources for estimating P×S effects and many are cited in this article.

## Conceptual Background

### Key Definitions

The variance partitioning approach defines P×S effects quantitatively, typically in repeated-measures experimental designs. Consider the design in which persons are exposed to the same situations and their anxiety in each is assessed (**Table 1**). There are three effects in this design: person, situation and Person × Situation interactions. Defining P×S effects requires that one first define person and situation effects.

**Table 1:** An example of a simple structure of a design to reveal Person × Situation effects.

	S_1_	S_2_	S_3_	Mean
**P_1_**	6	5	9	6.7
**P_2_**	5	7	5	5.7
**P_3_**	2	6	8	5.3
**Mean**	4.3	6.0	7.3	5.9

*Each of three persons is indicated by P_1_ – P_3_ and each of three situations is indicated by S_1_ – S_3_.*

Person effects indicate how much people differ from the grand mean in their levels of anxiety, averaged across situations. For example, Person 1 has higher anxiety than average, whereas Persons 2 and 3 have lower than average anxiety (**Table 1**). This effect reflects trait-like personality, as well as cross-situational consistency (Mischel, 1968) and is the traditional focus of personality psychology.

Situation effects indicate the extent to which situations differ from the grand mean in the extent to which they evoke anxiety, on average, across persons. For example, Situation 1 evokes lower anxiety in people than average, whereas Situations 2 and 3 evoke higher anxiety than average (**Table 1**). Situation effects are the typical focus of social psychology, but when estimated in repeated measures designs, also reflect within-person variation. Situation effects reflect normative variation in how persons’ anxiety, on average, ebbs and flow from one situation to the next. The effect is normative in that it captures people’s typical responses.

**FIGURE 1:**
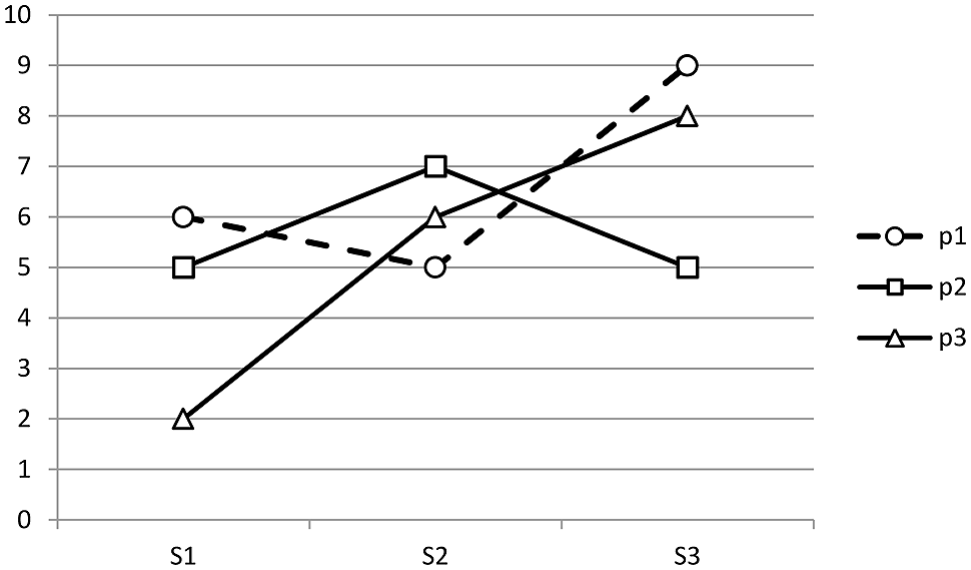
**P×S profiles from Table 1.** Each of three persons is indicated by P_1_ – P_3_ and each of three situations is indicated by S_1_ – S_3_.

P×S effects reflect how people differ in their profiles of anxiety across situations. For example, in **Table 1** and **Figure 1**, Person 1 has a different profile of anxiety across the three situations than does Person 2. Person 1 is highly anxious at funerals (S_3_), but not when giving speeches (S_1_) or when on first dates (S_2_). Persons 2 and 3 display a different pattern. P×S effects are defined quantitatively, and thus with clarity and precision: P×S = X_ij_ - P_i_ - S_j_ + M in which x_ij_ is person i’s score in response to situation j. The person’s mean score across all situations (person effects) is P_i_, S_j_ is the situation’s mean score across all persons (situation effects) and M is the grand mean. That is, Person 1 responds with more anxiety to funerals (x_ij_) than how she typically responds to situations on average (P_i_), and with more anxiety than people typically experience at funerals (S_j_). Phrased differently, funerals evoke unusually high anxiety in Person 1. Thus, like situation effects, P×S effects reflect within-person variation. However, P×S effects reflect within-person variation that is idiosyncratic to specific persons whereas situation effects reflect normative variation. Like person effects, P×S effects also capture individual differences. However, P×S effects reflect differences among persons in their profiles of responses to situations whereas person effects reflect differences among persons, on average, across situations.

### The Development of the Variance Partitioning Approach

The variance partitioning approach emerged first from Cronbach et al.’s (1972) G theory of test reliability. G theory describes how to conceptualize and estimate various substantive effects and sources of measurement error. Substantive effects are what investigators want to measure and error is everything else. The designs for estimating P×S effects are essentially similar to, and were derived from, designs used to estimate test reliability. Consider again **Table 1**. If one substitutes test items for situations, we have the classic design for estimating measurement error and the internal consistency of a test. Thus, person effects reflect the extent to which people differ in anxiety, on average across items. This is typically what investigators want to measure. Person × Item interactions are essentially Person × Situation interactions: the extent to which people have different profiles of responses across items. Within the context of measurement theory, P×I interactions indicate the extent to which differences among people depend upon the item (i.e., measurement error). Internal consistency reliability is based on the relative strength of person effects and Person × Item interactions, as well as the number of items in a test. The key insight was that the same procedures for estimating Person × Item effects (i.e., measurement error) could be used to estimate P×S effects. Endler and Hunt (1966, 1969) were the first to apply this insight when Cronbach, Endler, and Hunt were at the psychology department at the University of Illinois (Urbana/Champaign) in the early 1960s. These analyses were sufficiently advanced in their day that they had to be calculated with the university’s supercomputer.

The second major approach to studying P×S effects is the SRM (Warner et al., 1979; Kenny and La Voie, 1984; Malloy and Kenny, 1986; See Back and Kenny, 2010, for an accessible introduction). The SRM defines P×S effects in the same way as G theory, but applies to the special case in which other people are the situations and persons rate each other in a round-robin design. That is, instead of studying persons’ reactions to funerals, speeches and first dates, one studies reactions to Jenny, Richard, and Stephen. Treating people as situations is an important conceptual advance and the SRM also reveals effects not encountered in G theory. Social psychology typically examines classes of situations at a high level of abstraction that averages out the specifics. The hope is that what is learned about situations transcends the particulars, including the specific people who populate the situations (Kenny, 2006). Yet, funerals are very different depending upon whom the funeral is for and who is present. A funeral for the parent of a co-worker is one thing; a funeral for your parent is something else entirely. A funeral for your parent when you like your family is different from a funeral when you dislike your family. In other words, the SRM assumes that important determinants of the effects of situations are the specific people who populate the situation.

## Evidence for Strong PxS Effects

There are very strong P×S effects for many constructs, including family negativity (Rasbash et al., 2011), attachment (Cook, 2000), person perception (Park et al., 1997; Branje et al., 2003), aggression (Coie et al., 1999), psychotherapy (Marcus and Kashy, 1995; Lakey et al., 2008), romantic attraction (Eastwick and Hunt, 2014), and many more. The next section provides a more detailed review of P×S effects on anxiety, five-factor personality traits, perceived social support, leadership, and performance. The strength and replicability of P×S effects are impressive.

### Anxiety

Endler and Hunt (1966, 1969) applied the variance partitioning approach to P×S interactions in their seminal studies of anxiety. Endler and Hunt developed a questionnaire that assessed anxiety in specific situations. For example, “You are just starting off on a long automobile trip,” “You are getting up to give a speech before a large group,” and “You receive a summons from the police.” The data were analyzed as a Person × Situation design, as described previously (**Table 1**). Across 22 separate samples, P×S effects accounted for 17% of the variance in anxiety. Person effects accounted for 8% and situations accounted for 7%. That is, there were large effects whereby people had different profiles of anxiety across situations. For example, Richard might have more anxiety in response to receiving a summons than in making a speech; whereas Stephen might have more anxiety in making a speech than in receiving a summons. There were also substantial person effects whereby some people reported more anxiety, on average, across situations than did others. For example, Richard might be more anxious on average than are others. In addition, there were substantial situation effects whereby some situations (e.g., receive a summons) evoked more anxiety in people than did other situations, on average (e.g., beginning a car trip).

Ingraham and Wright (1987) also found very large P×S effects in anxiety using the SRM. They used a round-robin design in which each person in the sample rated every other person (i.e., situations) on how much anxiety the other evoked. Study 1 was composed of graduate students participating in a group therapy training experience and Study 2 was composed of group therapy outpatients. There were large P×S effects in both studies, accounting for 37% of the variance. For example, Richard experienced less anxiety with Stephen than (1) Richard typically experienced across people, and (2) Stephen typically evoked in people. That is, anxiety largely reflected the unique relationship between two people. For comparison, person effects accounted for 15% of the variance and situation effects (other people) accounted for only 3%. Very strong P×S effects on anxiety were recently replicated in round-robin studies of Marines and college roommates (Lakey et al., in press).

Thus, there are very large P×S effects in anxiety that are at least as large as trait anxiety. These findings replicate well, are found for nominal situations (e.g., funerals) as well when situations are other people.

### Five-Factor Traits

The five-factor model of personality has been widely influential as a standard framework for organizing personality characteristics, and the five traits are typically viewed as broadly generalizable across situations (Goldberg, 1990). Yet, people also have large idiosyncratic patterns in their levels of traits across situations. Van Heck et al. (1994) assessed neuroticism, extroversion, conscientiousness, agreeableness, and openness in a wide range of situations through self-report. Among Dutch and Italian college students, P×S, person, and situation effects were approximately equally strong, with each accounting for about 12% of the variance. Hendriks (1996) replicated these findings among Dutch college students and included peer reports as well. There were large P×S effects accounting for about 20% of the variance for each of the five traits. Hendriks (1996) also found person (≈20%) and situation effects (≈12%). Thus, although people differ in their typical levels of the five factor traits (person effects), people also have idiosyncratic profiles in their responses to situations. For example, Person 1 might have high levels of agreeableness during a quarrel and low levels when playing a game. Person 2 might show the opposite pattern. In summary, five factors traits show strong P×S effects.

### Perceived Support

Perceived support is the subjective judgment that friends and family would help during times of need and is a well-replicated marker of emotional well-being (Cohen and Wills, 1985; Barrera, 1986). Studying P×S effects for perceived support is essentially similar to studying anxiety or personality except that (1) the situations are people who provide support and (2) persons rate the supportiveness of providers rather than their own anxiety or personality. In a meta-analysis, P×S effects accounted for 62% of the variance in supportiveness (Lakey, 2010). Thus, the extent to which a person sees a provider as supportive is mostly idiosyncratic to the person. Phrased differently, the supportiveness of a provider reflects the unique relationship between the person and the provider. In addition to P×S effects, perceived support also reflects persons’ trait-like tendencies to see other people as supportive (27%) and a relatively small portion (7%) reflects agreement among persons that some providers are more supportive than others (situation effects). These findings have been observed when Ph.D. students rated faculty members (Lakey et al., 1996), elite youth athletes rated coaches (Rees et al., 2012), and medical residents rated clinical mentors (Giblin and Lakey, 2010). They have also been found when sorority sisters (Lakey et al., 1996), marines, college roommates (Lakey et al., in press), and nuclear family members rated each other (Branje et al., 2002; Lanz et al., 2004).

### Leadership

Leadership is a key concept in organizational behavior and theories vary widely in how leadership is conceptualized and studied. Yet, much research, theory and practice seems to reflect an implicit assumption that leadership is a trait-like characteristic of leaders (situations) that generalize across a range of followers (persons; Avolio et al., 2009). Variance partitioning studies of leadership provide a more nuanced approach. Most variance partitioning studies have used round-robin designs in which four- to five-person groups rate each other on leadership after completing a group task (Livi et al., 2008; Kenny and Livi, 2009). Tasks have included leaderless group discussions, thinking of essential items if stranded and thinking of ways to promote tourism. A recent meta-analysis found that 20% of leadership reflected P×S effects, 40% reflected leaders (situations) and 10% reflected followers (persons; Livi et al., 2008; Kenny and Livi, 2009). That is, the extent to which a given leader elicits a sense of leadership in followers partly reflected followers’ personal tastes. One sees this in presidential elections. Although one candidate is ultimately preferred by a majority of voters, there is also substantial disagreement among voters about which candidate is the best leader.

### Performance

An important question in applied psychology is how to improve people’s performance on tasks, such as typing, standardized tests, memory, vigilance, work performance, reading, and many others (Kanfer and Ackerman, 1989; Kluger and DeNisi, 1996). Research often focuses on how to train people (Kanfer and Ackerman, 1989; Kluger and DeNisi, 1996) and structure tasks (Gigerenzer and Goldstein, 1996) for optimal performance. Variance partitioning offers the unique focus on the extent to which performance is affected by the unique relationships among members of the work group. Consider three crewmembers operating a battle tank. The variance partitioning approach identifies three aspects of performance. Each crewmember has trait-like skill at the task (person effect) and each might elevate the performance of his other crew members (situation effects, as in leadership). In addition, the unique relationship between any two crewmembers might also elevate performance (P×S effects). If so, then in addition to selecting and training effective tank leaders (situations) and crewmembers (persons), tank teams might be selected so that the particular combination of soldiers (P×S effects) enhances performance beyond person and situation effects.

Recent research provides an example of identifying P×S effects on team performance (Woods et al., in press; Study 3). Groups of four strangers played a warfare video game that accommodated doubles play. Each person played the game with each of three teammates (situations) in a round-robin design and performance was assessed objectively as well as through self-reports. There were strong P×S effects in which a player’s performance depended upon the teammate with whom he was paired, accounting for 74% (self-rated) and 35% (objective) of the variance. For example, Ken might display unusually good performance when paired with Matt, than when paired with Bill, beyond Ken’s trait-like skill and Matt’s ability to elevate performance in his teammates. There were also strong person effects in that some players had higher skill than did others, accounting for 23% (self-rated) and 63% (objective) of the variance in performance. There were no effects whereby some teammates elevated the performance of all other teammates (situations, cf. leadership).

Other investigators have documented P×S effects for memory performance following training (Gross et al., 2009, 2015). Persons heard presentations from different trainers (stimuli) and were tested on retention. There were significant P×S effects on memory following training, in that a person’s memory for training depended, in part, on which trainer presented the material. For example, Person 1 might have unusually good memory for Trainer 1’s presentation than for Trainer 2 or 3. Person 2 might show a different pattern.

Thus, there is emerging evidence for strong P×S effects on task performance. It would be straightforward to apply the variance partitioning approach to a wide range of human performance problems.

To conclude this section, very strong P×S effects have been observed for a wide range of constructs, including anxiety, five-factor personality, perceived support, leadership and task performance. Given the replicability, strength and broad generality of P×S effects, the variance partitioning approach should be widely used in many research areas. This does not seem to have happened. Why not?

## Developing a Psychological Understanding of PxS Effects

Although strong P×S effects are ubiquitous, it has been hard to make sustained progress in understanding them. Time and again, large P×S effects are observed for a construct and no further progress is made. After estimating the size of P×S effects, it has not been clear how to move forward.

How can investigators develop a psychological (versus purely statistical) understanding P×S effects? This is a special case of the general problem of how to develop a psychological understanding of anything. Cronbach and Meehl’s (1955) seminal work on construct validity provides the key answer. The solution is merely to apply the general strategy of construct validation to the special case of P×S effects. This involves simply developing the nomological network for the P×S aspect of a construct, including (1) establishing the other constructs to which the P×S aspect is related (2) identifying mechanisms for the P×S aspect and (3) forecasting future outcomes from the P×S aspect.

According to Cronbach and Meehl (1955), construct validity is built by developing an understanding of a new construct’s empirical properties (i.e., its nomologial network). In personality research, this primarily involves understanding the new construct’s correlations with other constructs. Over time, well-replicated links between the new construct and other constructs are established. Some of the links fit well with the rudimentary theory; others do not. The rudimentary theory is revised in light of these findings and new studies are devised to test the revised theory. Thus, one begins an iterative series of empirical studies and theory revision. In this way, one develops the validity of a new construct by pulling up by one’s bootstraps.

Here is an example of how this process has worked for perceived social support. Perceived support measures were developed to assess the extent to which friends and family helped with stressors (Barrera, 1986). The word “perceived” was used only to acknowledge that the measures relied upon self-report. Yet, perceived support was hypothesized to reflect the actual help that friends and family provided to promote coping and thereby protect persons from the harmful effects of stress. As expected, people with high perceived support had better emotional well-being than did people with low support (Cohen and Wills, 1985; Barrera, 1986). Yet, it was not long before other findings cast doubt on the original theory. For example, perceived support was not very closely related to support actually received from family and friends (Barrera, 1986), and support received was not consistently linked to better emotional well-being (Barrera, 1986; Finch et al., 1999; Bolger et al., 2000). Instead, perceived support was much more closely linked to perceptions of providers as similar to recipients in attitudes and values (Lakey et al., 2002). In addition, most of perceived support’s links to emotional well-being did not involve stress buffering, but occurred regardless of the presence of stress (Lakey and Orehek, 2011). Such findings were inconsistent with the original theory, led to additional empirical studies and the development new theories (e.g., Uchino, 2009; Lakey and Orehek, 2011). Some research findings will not fit the new theories, and this iterative process will continue. Thus, one develops a psychological understanding of perceived support.

How does one apply construct validity to P×S effects? This question seems to have been the sticking point in making progress, and the solution is both technical and conceptual. Building construct validity requires linking constructs to other constructs, but P×S effects are represented as profiles of scores across situations (**Figure 1**). How does one establish a nomological network for profiles of scores? Cronbach et al. (1972) provided the answer with multivariate generalizability analyses (see Strube, 2000, for an accessible introduction). The key insight is that since P×S aspects are represented as profiles, all other constructs must also be represented as profiles. In addition, the profiles must be commensurate. That is, if the P×S aspect of a construct is represented as a profile across five situations, the P×S aspect of another construct must also be represented across the same five situations.

Thus, it is not meaningful to correlate the P×S aspect with the trait aspect of a construct because they are represented incommensurately. As depicted in **Table 1**, each person has a profile of anxiety in the three situations. Each person also has an anxiety score averaged across the three situations (the person aspect). Estimating a correlation between trait anxiety and each person’s profile requires mapping the three P×S profile scores onto the single person score. Of course, this cannot be done meaningfully, in part because each P×S score has already had the person aspect of anxiety removed. Moreover, there is more information in a three-score profile than can be contained in a single person score. Using a questionnaire measure of trait anxiety does not solve the problem, because we are still left with the issue of mapping three bits of information onto a single bit. Thus, one cannot explain the P×S aspect of anxiety in terms of the five factor traits, unless the traits are also expressed as profiles. It is straightforward to represent the five factors as profiles (Van Heck et al., 1994; Hendriks, 1996), but doing so changes their meaning. At minimum, the P×S aspects of the five factors are no longer traits.

Historically, a major obstacle in applying Cronbach et al.’s (1972) insight was the lack of computer programs for conducting the analyses. Kenny (unpublished computer program) developed a program for round-robin analyses and Brennan (2001) developed a program for more typical G designs. In addition, such analyses can be done with structural equations and multilevel modeling (Biesanz, 2010; Ackerman et al., 2015).

### Developing Nomological Networks for P×S Effects: The Case of Perceived Support

Perceived support research provides an example of developing the nomological network for the P×S aspects of constructs. A core finding in perceived support research (Cohen and Wills, 1985; Barrera, 1986) is that perceived support is linked to emotional well-being. Thus, it is important to determine that this link occurs for the P×S aspects of support and well-being specifically.

**FIGURE 2:**
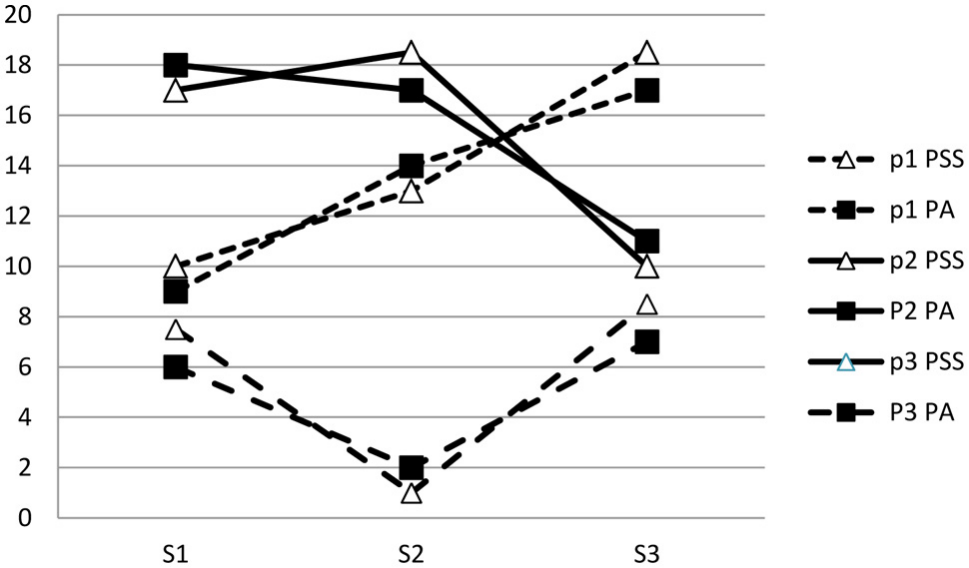
**P×S profiles of supportiveness (PSS) covary with P×S profiles of positive affect (PA).** Each of three persons is indicated by P_1_ – P_3_ and each of three situations is indicated by S_1_ – S_3_.

Investigators have studied persons in the laboratory as they had conversations with the same support providers (situations), on multiple occasions (Neely et al., 2006; Veenstra et al., 2011). After each conversation, persons rated their positive and negative affect during the conversation, as well as the supportiveness of the provider. Independent observers also rated the conversations in Neely et al. (2006). Both studies found that the P×S aspect of perceived support was linked to the P×S aspects of high positive, and low negative affect. That is, when a provider evoked unusually high positive or low negative affect in a person, the person saw the provider as unusually supportive. That is, each person’s profile of affect across providers covaried with her profile of supportiveness across the same providers (**Figure 2**).

Most social support research is field research and the variance partitioning approach can easily be applied to field contexts. For example, in one study, participants rated their perceived support and affect typically evoked by important support providers (Lakey et al., in press). In round robin designs, marines and college roommates rated each other. As found in laboratory studies, the P×S aspect of supportiveness was linked to the P×S aspect of affect. That is, when a provider evoked unusually high perceived support in a person, the provider also evoked unusually favorable affect.

These examples show that establishing the nomological network, and hence the construct validity of the P×S aspect of a construct is essentially the same as for any other construct. The key difference is that correlations must be estimated for the P×S aspects of constructs specifically, and thus studies must be designed to isolate P×S aspects.

If one wants to understand the P×S aspect of a construct, one cannot use conventional research methods. Consider a conventional study in which persons rate the supportiveness of their social networks and their own emotional well-being. A typical finding is that perceived support is linked to emotional well-being. Unfortunately, the design cannot reveal the extent to which the link between perceived support and emotional well-being reflects, (1) the trait-like tendencies of persons to see everyone as supportive and to experience well-being (person effects), (2) persons’ good fortune to be surrounded by providers who evoke a sense of support and well-being in nearly everyone (situation effects), or (3) the unique relationships between persons and providers in which the provider who elicits unusually high support in a person also elicits unusually good emotional well-being (P×S effects). The psychological meaning of these correlations differs dramatically depending upon which aspect of support the correlations reflect. The correlation between perceived support and emotional well-being, estimated with conventional methods, could reflect any one of the three effects, or some unknown combination of the three.

### Identifying Mechanisms for P×S Effects

Part of developing a nomologial network is identifying the mechanisms by which constructs are linked, but in the P×S research just described, no mechanisms were identified. We learned that when a person saw a provider as unusually supportive, the provider also evoked unusually favorable affect, but the studies did not indicate how this occurred. For example, how did Person 1 arrive at a judgment of Provider 1’s supportiveness that was different from how Person 1 typically sees other providers, and different from how Provider 1 is typically seen?

Lutz and Lakey (2001) hypothesized that the P×S aspect of perceived support emerges, in part, because persons weigh information about providers (situations) differently when judging support. Persons use information about providers’ personality (e.g., agreeableness and emotional stability) to judge providers’ supportiveness (Lakey et al., 2002). Lutz and Lakey (2001) tested the hypothesis that persons weigh these traits differently. In two studies, persons were presented with descriptions of over 100 providers who differed in their five-factor personality profiles. For example, one provider was described as “self-conscious, not self-assured, somewhat reliable, very literary, not tender-hearted.” The investigators could derive regression equations that described how each person used information about providers’ personality to judge providers’ supportiveness. As predicted, there were significant differences in how persons’ weighed personality traits to judge supportiveness.

**FIGURE 3:**
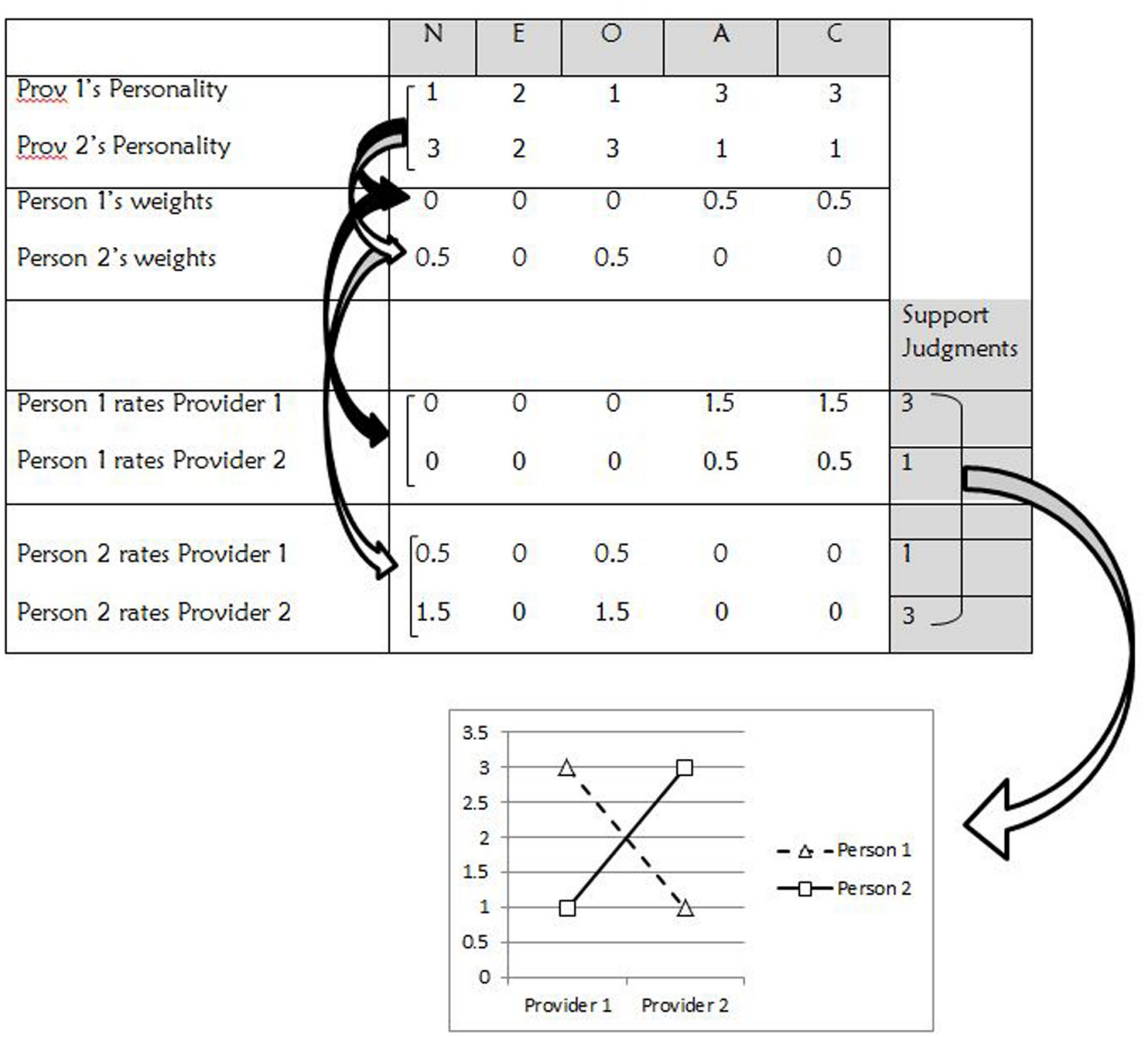
**P×S effects emerge when persons weigh providers’ traits differently in forming support judgments.** N = neuroticism; E = extroversion; O = openness; A = agreeableness; C = conscientiousness.

To see how these differences can explain P×S effects, consider the case depicted in **Figure 3** in which Persons 1 and 2 rate Providers 1 and 2. Providers 1 and 2 have different five-factor profiles. For example, Provider 1 has high agreeableness and conscientiousness and Provider 2 has high neuroticism and openness. Person 1 and Person 2 weigh provider traits differently in rating supportiveness. Person 1 weighs provider agreeableness and conscientiousness heavily and Person 2 weighs neuroticism and openness heavily. Each person’s judgment of each provider is determined by (1) multiplying each provider’s personality trait score by (2) the weight typically used by each person to judge support from the trait. For example, Provider 1’s agreeableness score of 3 is weighed by 0.5 by Person 1, but weighed by 0 by Person 2, contributing to disagreement about Provider 1’s supportiveness. Thus, when persons weigh provider traits differently in judging support, persons disagree about the supportiveness of the providers, resulting in P×S profiles. This mechanism is essentially similar to Mischel and Shoda’s (1995) hypotheses that links among mediating units translate encoded information about situations to each person’s unique profiles of responses to situations.

### Forecasting Important Outcomes for the P×S Aspects of Constructs

An important part of the validity of a construct is that it can forecast future outcomes. For example, the construct validity of conscientiousness is supported by the fact that job applicants’ conscientiousness scores forecast their subsequent job performance (Oh et al., 2011). Forecasting the P×S aspects of constructs is a simple extension of establishing a nomological network among P×S aspects: P×S profiles from Time 1 are used to forecast P×S profiles at Time 2. What follows are two examples of forecasting the P×S aspects of constructs.

There are large P×S effects for students’ (persons) evaluations of instructors’ (situations) teaching (Gross et al., 2009, 2015). That is, Student A might find Instructor A to be more effective than Instructor B, but Student B might have the opposite opinion. Given the large size of P×S effects, it might be useful to forecast which students will find which instructor especially effective, so that specific instructors could be recommended to specific students to optimize instruction.

Gross et al. (2015) tested this concept by developing brief videos of instructors’ teaching. The teaching trailers (cf. movie trailers) were shown to a group of students in three large college classes at the beginning of the semester. Students rated the effectiveness of each instructor’s teaching in response to the trailer. Later in the semester, students heard hour-long lectures from each of the instructors and rated the effectiveness of each. Forecasting the P×S aspect of teaching effectiveness involved mapping each student’s profile of responses to the trailers at Time 1 to his profile of responses to lectures at Time 2. In fact, Gross et al. (2015) could accurately forecast the instructors that specific students found unusually effective.

A second example of forecasting future outcomes for P×S profiles comes from social support research. Given the strong P×S effects on perceived support, one approach to intervention is to assign specific support providers to specific persons, such that unusually supportive relationships emerge. Such an approach requires the technology to forecast which person will see which provider as uniquely supportive. Veenstra et al. (2011) forecasted the P×S aspect of supportiveness from brief conversations between persons and providers (situations). That is, a person’s reaction to a stranger from a brief conversation forecasted the extent to which the person ultimately saw the former stranger as unusually supportive weeks and months later. Veenstra et al.’s (2011) analytic approach was the same as in Gross et al. (2015). From the first conversation (Time 1), each person had a profile of scores across the providers. Each person also had a profile of scores across the providers at Time 2. Forecasting P×S effects from Time 1 to Time 2 involved calculating the correlation between the Time 1 profiles and the Time 2 profiles.

The variance partitioning approach to P×S forecasting just described is essentially similar to that described by Shoda et al. (1994), except that the variance partitioning approach is simpler. Shoda et al. (1994) observed four types of children’s behavior (e.g., prosocial, whining) across five types of situations (e.g., peer approaches, adult warns), over two time periods. For each child, Shoda et al. (1994) constructed profiles of responses for each behavior across the five situations. In calculating the profiles, each child’s person score and each situation’s score was removed. Thus, the profiles were identical to P×S profiles. Shoda et al. (1994) found that P×S profiles at Time 2 could be forecasted from P×S profiles at Time 1. However, this approach requires (1) calculating profiles for each person, (2) calculating correlations between profiles at Time 1 and Time 2 for each person and then (3) taking the average of the correlations across persons. In contrast, the variance partitioning approach achieves these steps in a single, ANOVA-like analysis.

To summarize, this section described how to establish the construct validity of the P×S aspects of constructs. In principle, it is no different from establishing the validity of any construct. In tandem with theory development, one establishes a network of associations to other constructs. This process differs for P×S aspects only in that constructs are represented as profiles rather than as single scores. Yet, isolating the P×S aspects likely requires some re-conceptualization of the construct. For example, neuroticism is typically viewed as a trait that it is stable across situations and time. Yet, P×S neuroticism is not a trait, in that it is not stable across situations. By extension, mechanisms that are geared to explain the trait-like aspect of neuroticism (e.g., chronically accessible constructs or catecholamine dysfunction) might not translate well to P×S profiles. Thus, theories of the P×S aspect of neuroticism would need to focus on mechanisms that can take into account how different situations evoke different levels of neuroticism in different people.

## PxS Effects can Contribute to Theory

The variance partitioning approach to P×S effects can make an important contribution to theory development. The approach can increase conceptual clarity by requiring the theory to be explicit about whether the core constructs are P×S, person or situation effects. If the theory can be made explicit, the variance partitioning approach provides guidance about research designs to test the theory with greater precision and recommends approaches to intervention. Examples from social support research will be used to illustrate these points.

Until recently, social support theory has been vague about whether perceived social support reflects P×S, person or situation effects. Most social support theory implies that perceived support reflects situation (provider) effects such that persons agree that some providers are more supportive than others and consensually supportive providers have beneficial effects on persons’ emotional well-being (Thoits, 1986). Yet, there is a minority view that perceived support is a property of persons (Sarason et al., 1986; Lakey and Cassady, 1990; Uchino, 2009). That is, some persons are predisposed to see providers as supportive and to have good emotional well-being. Recent theory conceptualizes perceived support as a P×S interaction (Lakey and Orehek, 2011). Conventional research designs have been unable to discriminate among these interpretations. Greater conceptual clarity on the nature of perceived support is helpful.

One would design studies differently depending upon whether one conceptualized perceived support as an aspect of the person, the provider (situation), or a P×S interaction. As described previously, to capture the P×S aspect of perceived support, one must isolate each person’s profile of supportiveness (and other constructs) across a number of providers, while removing person and situation effects. This typically requires a repeated-measure experimental design in which at least subsets of persons rate the same providers. To capture the person aspect, one should average perceived support (and other constructs) across many providers, situations, and time. To capture provider effects, one should have many providers rated by many persons; providers (instead of persons) should be treated as subjects. Ironically, although most social support research at least implicitly conceptualizes support as an aspect of providers, almost no research has used designs that capture provider effects specifically.

The variance partitioning approach also provides useful guidance about how to help people change. One would approach intervention very differently depending upon whether one wanted to target the P×S, person or situation aspect. Social support interventions provide an example. Most interventions have been designed to work through provider effects. Thus, a set of providers are selected by project staff and made available to persons. This assumes that selected providers will be seen as supportive by nearly all persons and the providers will evoke better emotional well-being in nearly everyone (Heller et al., 1991). However, if one wanted to influence the person aspect of perceived support, interventions should attempt to change persons. For example, training persons in social skills and in resisting cognitive biases might alleviate tendencies to see everyone as unsupportive (Brand et al., 1995). Interventions to modify the P×S aspect of supportiveness would pair persons with providers such that unusually supportive relationships emerged (Lakey and Orehek, 2011).

To summarize this section, variance partitioning approaches can contribute to theory development by providing (1) greater conceptual precision in descriptions of core constructs, (2) guides to study design to test theories with greater precision, and (3) guides to intervention. Perceived support served as an example in this section, but the basic principles could be extended to a wide range of constructs. For example, to what extent is adult romantic attachment a feature of the person (he is insecure with everyone), a feature of the situation (she elicits insecurity in everyone) or an aspect of P×S effects (he is uniquely insecure with her)? To help him develop more secure attachment, should he seek psychotherapy to change his predispositions or get a different romantic partner? If he gets a different romantic partner, should he look for a partner who elicits security in everyone or a partner who elicits high security in him uniquely? As another example, is leadership a property of the leader (stimulus), the unique relationships (P×S) among specific leaders and followers, or the dispositions of followers (persons) to see everyone as good leaders? Training people to become better leaders assumes implicitly that leadership is a property of leaders and that people can learn leadership qualities that are broadly generalizable across followers and contexts. Alternatively, one might select leaders who are well-matched with the followers in a particular organization, or a leader might elect to lead an organization composed of dispositional followers.

## Challenges Facing Variance Partitioning Approaches

There remain important challenges to understanding the P×S aspect of within-person variation. These include reducing the information density of P×S profiles, forecasting P×S profiles in response to novel stimuli and studying contexts in which persons do not encounter the same situations.

### Reducing the Information Density of P×S Profiles

In variance components research, P×S profiles are represented so that each person is a level of a person factor and each situation is a level of a situation factor (**Table 1**), as described previously. This is an information-dense representation, as it requires large amounts of information about situations and persons. Even in a small study with 10 persons and 10 situations, 100 cells would be needed to represent each person’s P×S profile. The information density of such designs can easily exceed software capacity and investigators’ working memories. A simpler representation would be to classify persons and situations into categories. For example, in the 10 × 10 design just described persons and situations could each be classified into one of two categories, reducing the 100-cell design to four cells (2 × 2). A simpler representation would be preferable, as long as it could explain variance nearly as well as the more information-dense design. Yet, as described momentarily, there is no guarantee that P×S effects revealed in an information-dense design will be captured in a simpler design.

Most individual differences research uses only simple representations in the search for P×S effects. For example, research on depression and negative life events classified persons as high in dependency or self-criticism and classified life events as relevant to either interpersonal or achievement concerns (Hammen et al., 1985; Coyne and Whiffen, 1995). Dependent people were predicted to respond to interpersonal events (e.g., marital conflict) and self-critical people were predicted to respond to achievement events (e.g., failing a training program). Although initially promising, the work has not yielded very replicable findings (Coyne and Whiffen, 1995). One possibility is that there are, in fact, P×S effects in how people respond to events, but the research represented P×S profiles too simply to capture the effect.

**Table 2** uses simulated data to illustrate how P×S effects in a high-density design might not be captured in a simpler design. Panels A and B include exactly the same data points and differ only in how they are arranged. Both panels include a high-density design as well as a simpler design. When analyzed as a high-density design, both panels yield very strong P×S effects with no person or situation effects. How well does the simpler design capture the P×S effect revealed in the high-density design? In Panel A, the simpler design accounts for all of the P×S effect. All dependent persons respond with increased depression to interpersonal events, but not to achievement events. All self-critical persons respond to achievement events, but not to interpersonal events. However, in panel B, the high-density P×S effect is not captured by the simpler design at all. Of course, if the simpler design captures the P×S effect well (Panel A), there would be no need to use the information-dense design. Yet, if the simpler design does not capture the P×S effect (Panel B), one would have to rely upon the high-density design. If one had only the simple design, one might incorrectly conclude that there were no P×S effects. Unfortunately, the simple design is what psychologists studying Person × Situation interactions typically have. If one happens to choose the right classification scheme, one will find a P×S effect. However, it might be better to start with the high-density design to see if a P×S effect is present. Then, one can figure out how to represent the effect with a simpler classification scheme.

**Table 2:** P×S effects in a high-density design captured well **(A)** and poorly **(B)** by a simpler design.

		Situation class			
		Interpersonal		Achievement	
Person class	Persons	Situations			
		S_1_	S_2_	S_3_	S_4_
**(A)**					
Dependent	P_1_	4	4	2	2
Dependent	P_2_	4	4	2	2
Self-critical	P_3_	2	2	4	4
Self-critical	P_4_	2	2	4	4
**(B)**					
Dependent	P_1_	2	2	4	4
Dependent	P_2_	4	4	2	2
Self-critical	P_3_	2	4	4	2
Self-critical	P_4_	4	2	2	4

*Each of four persons is indicated by P_*1*_ – P_*4*_ and each of three situations is indicated by S_*1*_ – S_*4*_.*

The variance partitioning approach is well-suited to analyze how well a simpler design can capture P×S effects revealed by a high-density design. Note that in **Table 2**, persons are nested within the dependent or self-critical class and situations are nested within the interpersonal or achievement class. If the simple design can capture a P×S effect present in the high-density design, we should see that the variance accounted for the P×S effect in the high-density design shifts to the Dependent/Self-critical × Interpersonal/Achievement interaction when the nesting factors are added.

It might be the case that many P×S profiles revealed in high-density designs cannot be adequately captured by simpler designs. If so, one will have to learn how to study P×S profiles in information-dense designs. Fortunately, the variance partitioning approach provides a way of conducting research with high-density designs. As described earlier in a different context, one can characterize the kinds of situations that elicit unusually strong reactions (P×S effects) in specific persons. For example, providers (situations) who evoke unusually high positive affect in persons are seen by persons as unusually similar to themselves, agreeable, supportive, eliciting good ordinary conversation as well as sharing activities (Lakey et al., 2004, in press). If an investigator does not want to rely on persons’ subjective judgments to characterize situations, one could study more objective indicators. For example, the provider who evoked unusually high objective task performance in a person also evoked unusually few automatic negative thoughts and high self-rated performance (Woods et al., in press; Study 3).

### Forecasting P×S Profiles for Novel Situations

How can we forecast a person’s profile of responses to situations he has never faced? The approach to forecasting P×S profiles described by Veenstra et al. (2011) and Gross et al. (2015) do not apply to this question because their approach requires that persons have had brief exposures to the situations. Here, the prediction problem is when there is no prior exposure.

One approach would be to determine for each person how she weighs information about situations and then apply those weights to generate predictions about reactions to new situations. Thus, a regression model would be developed for each person. To forecast how a person would respond to novel situations, one would obtain descriptions of each novel situation on the same dimensions used to develop each person’s regression model. For example, Lutz and Lakey (2001) developed individual regression models to describe how people used the five factor traits to judge provider supportiveness. To forecast judgments of novel providers, one would need descriptions of the providers’ five-factor traits. Applying the persons’ weights to the providers’ features would generate predictions of how each person would react to each provider. This approach is commonly used in commercial recommender systems such as Pandora. In the Pandora system, raters evaluate songs on a number of dimensions. Users (persons) indicate the songs they like. From user ratings, weights are presumably derived about how persons use the dimensions to judge songs. These weights are presumably used to predict reactions to new songs. Pandora is a proprietary system, and thus the details of the approach, as well as how well the approach predicts outcomes, are not explicit.

Although this approach should work in principle, there will be challenges in making such predictions with high precision. For example, how well will raters’ descriptions of new situations generalize to each person’s perceptions of the situations? We might know that a person weighs agreeableness heavily in judging providers. We might also know that observers have rated a novel provider as agreeable. In this case, we would forecast that the person would see the provider as supportive. However, the accuracy of the prediction will be limited by how well the observers’ ratings generalize to the person’s perception of the provider as agreeable, especially after the person has gotten to know the provider. If the person ultimately sees the provider as disagreeable, the original prediction based on observers’ descriptions of the provider will be inaccurate. There is good reason to believe that generalizing observers’ ratings to persons will introduce important imprecision, as inter-rater agreement about the personality traits of providers typically account for only about 30% of the variance (Kenny et al., 1994). Nonetheless, the variance partitioning approach provides the analytic tools for addressing these questions.

### Sometimes Situations are Nested within Persons

Throughout this article, the assumption has been that persons are exposed to the same situations. Yet often, important situations are encountered by only a few people. That is, situations are nested within persons. For example, one has a small number of parents, and except for one’s siblings, these parents are not shared with other people. One solution is to study only persons who encounter the same situations. Yet such designs exclude many people and situations. Another solution is the one-with-many design (Kenny et al., 2006). In one such design, situations (the many) are nested within persons (the one). For example, Lakey and Scoboria (2005) studied persons’ reactions to their mothers, fathers and closest friends and no one in the sample shared the same parents and closest friends. In such a design, it is not possible to separate P×S effects from situation effects. This is because P×S effects cannot be defined without first defining situation effects and situation effects require that at least sub-sets of participants encounter the same situations. Thus, P×S effects are confounded with situation effects. In another example, Marcus et al. (2011) studied therapy patients (the many) who each rated his therapist (the one). This design can isolate therapist (situation) effects, but person and P×S effects are confounded because no patients rated the same therapists, and no patients rated multiple therapists.

Designs that confound P×S effects with other effects can be a serious problem if one wants to understand P×S effects. However, the problem might not be so serious under some circumstances. For example, situation effects are very small compared to P×S effects for perceived support (Lakey, 2010) as well as for negative affect (Ingraham and Wright, 1987; Lakey et al., in press). Thus, for these constructs, the confounded (situation + P×S) effect in one-with-many designs primarily reflects P×S effects. Yet there is no guarantee that this will occur for other constructs. A given construct might primarily reflect situation effects (e.g., leadership), in which case one-with-many designs would be useless for understanding P×S effects. Thus, one must estimate the relative strength of situation and P×S effects in fully crossed designs before confidently interpreting the results of one-with-many designs. Still, for some constructs, the one-with-many design can be a useful tool for understanding P×S effects, especially since one-with-many designs are typically much easier to execute than round-robin studies.

### Is it Always Necessary to Develop Separate Nomological Networks for P×S Effects?

As described previously, one develops the construct validity of the P×S component of a construct by developing its nomologial network. One problem is that studies that isolate P×S components are typically more difficult to execute than are more conventional designs. Couldn’t one use more conventional research designs to estimate the P×S nomological network? One could do this, and it might work under some circumstances. However, one runs the risk of mistakenly assuming that a correlation between constructs occurs for the P×S component when it does not. There are several examples in which aspects of the nomological networks for constructs differed depending upon the variance component that was studied. Examples include adult romantic attachment (Barry et al., 2007), enacted support (Lakey et al., 2010), capitalization support (Shorey and Lakey, 2011) perceived support (Lakey et al., in press) and the link between positive and low negative affect (Lakey and Scoboria, 2005; Barry et al., 2007; Shorey and Lakey, 2011). Thus, one cannot know that a correlation between constructs occurs for a given component until one conducts studies that isolate the component.

## Summary and Conclusion

If the reader is interested in Person × Situation interactions and is willing to take the variance partitioning approach, there is a very good chance that he will be rewarded with very large P×S effects for nearly any psychological construct he chooses to study. Moreover, with some modification, he can apply the same construct validation procedures used for personality more generally to develop a psychological understanding of the P×S aspects of constructs. The variance partitioning approach can add increased precision to theory by defining with greater clarity key aspects of constructs. Understanding whether the key constructs are features of the person, the situation, or P×S interactions will help him design studies to test theory with greater precision, and will provide a useful guide for training and intervention.

## Conflict of Interest Statement

The author declares that the research was conducted in the absence of any commercial or financial relationships that could be construed as a potential conflict of interest.
